# 185. *Pseudomonas aeruginosa* (PA) Bacteremia and Patient Outcome during COVID-19 Pandemic

**DOI:** 10.1093/ofid/ofad500.258

**Published:** 2023-11-27

**Authors:** Renuga Vivekanandan, Kelsey Witherspoon, Christopher J Destache

**Affiliations:** Creighton University, Omaha, Nebraska; Creighton University School of Medicine, Omaha, Nebraska; Creighton Univ. Sch of Pharm & Allied Health Prof, Omaha, Nebraska

## Abstract

**Background:**

Pseudomonas aeruginosa (PA) bloodstream infection is associated with poor clinical outcomes. The purpose of this study was to evaluate treatment efficacy and patient outcomes from different antimicrobials used for treating PA bacteremia during 2020-2022.

**Methods:**

This was a retrospective review of 2020-2022 hospitalized patients with PA bacteremias at our health system. Hospitalized patients from January 2020 - July 2022 with culture-positive PA were identified. Data collected included demographics, hospitalization and drug treatment length, SARS-CoV-2 infection, and need for vasopressors. Antibiotics used to treat PA, including dose, interval, and MIC data were evaluated. Finally, admission APACHE II score and hospital mortality were collected. Data were analyzed by SPSS. Data are presented as mean ± SD or percentage. *Apriori* significance was p ≤ 0.05.

**Results:**

A total of 111 PA bacteremias occurred in 2020-2022, 65% of patients were male. Mean (± SD) age was 68 ± 13 years and weight were 88.2 ± 25 kg. Hospitalization length and duration of antibiotic therapy averaged 12 ± 13 days and 7.1 ± 5.9 days, respectively. Majority were treated with piperacillin-tazobactam (45%) or cefepime (44%). Median cefepime MIC was 2.0 (range 0.5-8). Thirty-seven (33%) PA-infected patients expired. Significantly more 7 of 17 (41%) cefepime patients died receiving 4G/d (1Gq6h) compared to 1 (9%) of 11 receiving 6G/d (2Gq8h), p< 0.001. Significant correlation between mortality and ICU status or mechanical ventilation (p< 0.001), respectively. No correlation between mortality and renal failure, SARS-CoV-2 co-infection, or vasopressor use. APACHE II score was significantly higher for expired patients (survived 13.9 ± 5.7 compared to 22 ± 8.3 in expired group, p< 0.001).

**Conclusion:**

ICU status and mechanical ventilation significant reduced survival. Cefepime 4G/d resulted in significantly higher mortality.

**Disclosures:**

**Christopher J. Destache, Pharm. D.**, BioMerieux, Inc: Advisor/Consultant

